# An Unusual Cause of Sepsis: Infected Iliofemoral Junction False Aneurysm following Extracorporeal Membrane Oxygenation

**DOI:** 10.1055/s-0040-1715467

**Published:** 2021-03-24

**Authors:** Bruno Pelissier, Guillaume Lebreton, Pascal Leprince, Pierre Demondion

**Affiliations:** 1 Department of Cardiac Surgery, Sorbonne Université, Pitié-Salpêtrière Hospital, Assistance Publique-Hôpitaux de Paris, Paris, France

**Keywords:** infected femoral artery false aneurysm, ECMO, heart transplantation, cryopreserved arterial homograft

## Abstract

The article presents an unusual cause of sepsis happening several weeks after heart transplant (infected iliofemoral junction false aneurysm) requiring iliofemoral reconstruction with arterial homograft by both retroperitoneal and inguinal approaches combined with Sartorius myoplasty.

Here we present a case of 61-year-old male patient referred for an unusual cause of sepsis after heart transplant. Due to severe ischemic cardiomyopathy, the patient underwent a left ventricular assist device implantation in January 2018. In January 2019, orthotopic heart transplantation was performed with concurrent percutaneous venoarterial extracorporeal membrane oxygenation (ECMO) implantation for a primary graft dysfunction during 6 days. Seven weeks after the heart transplant, while there was no visible clinical sign of infection, especially at the ECMO cannulation site, recurrent bacteremia ( *Proteus mirabilis* ) warranted a computed tomography (CT) and positron emission tomography scans, revealing a hypermetabolic focus on a false aneurysm of the left iliofemoral junction ( Fig. 1 ). Intraoperative findings confirmed a left infected iliofemoral junction false aneurysm ( Fig. 2 ). Surgical therapy consisted of an iliofemoral cryopreserved arterial homograft by both retroperitoneal and inguinal approaches combined with Sartorius myoplasty ( Fig. 3 ). We totally removed the false aneurysm and the infected artery ( Fig. 2 ). Peroperative bacteriological samples were positive to *P. mirabilis* , indicating a 2-week meropenem–vancomycin therapy. As the infection was not severe, we did not change the immunosuppression protocol. The wound was healed by secondary intention using vacuum-assisted closure therapy. At present, the patient is doing well. Postoperative CT did not show any anastomotic pseudoaneurysms. Wounds are clean and no skin infection is noted.

**Fig. 1:**
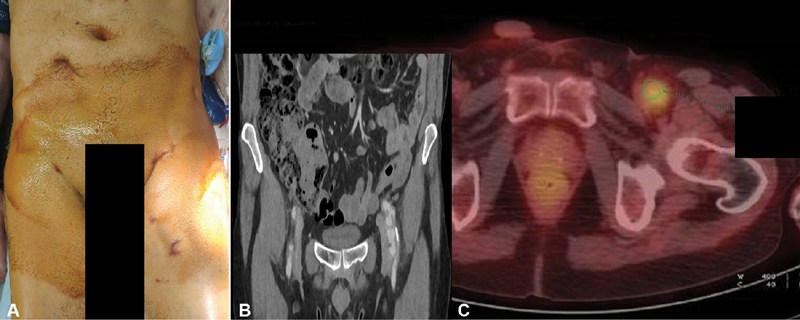
Extracorporeal membrane oxygenation cannulation site ( **A** ), computed tomography ( **B** ), and positron emission tomography scan ( **C** ), revealing a hypermetabolic focus on a false aneurysm of the left iliofemoral artery.

**Fig. 2:**
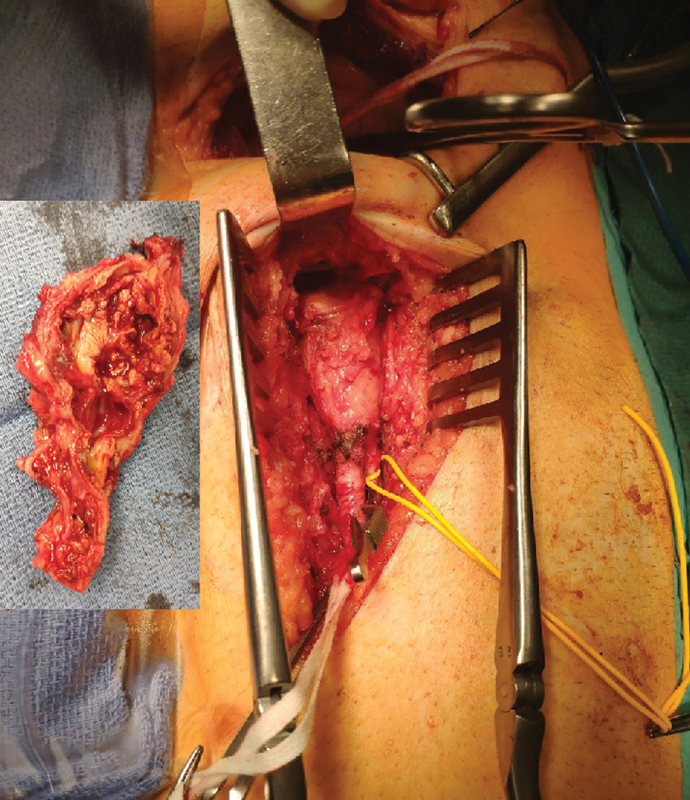
Intraoperative view: left iliofemoral artery false aneurysm infection by both retroperitoneal and inguinal approaches.

**Fig. 3:**
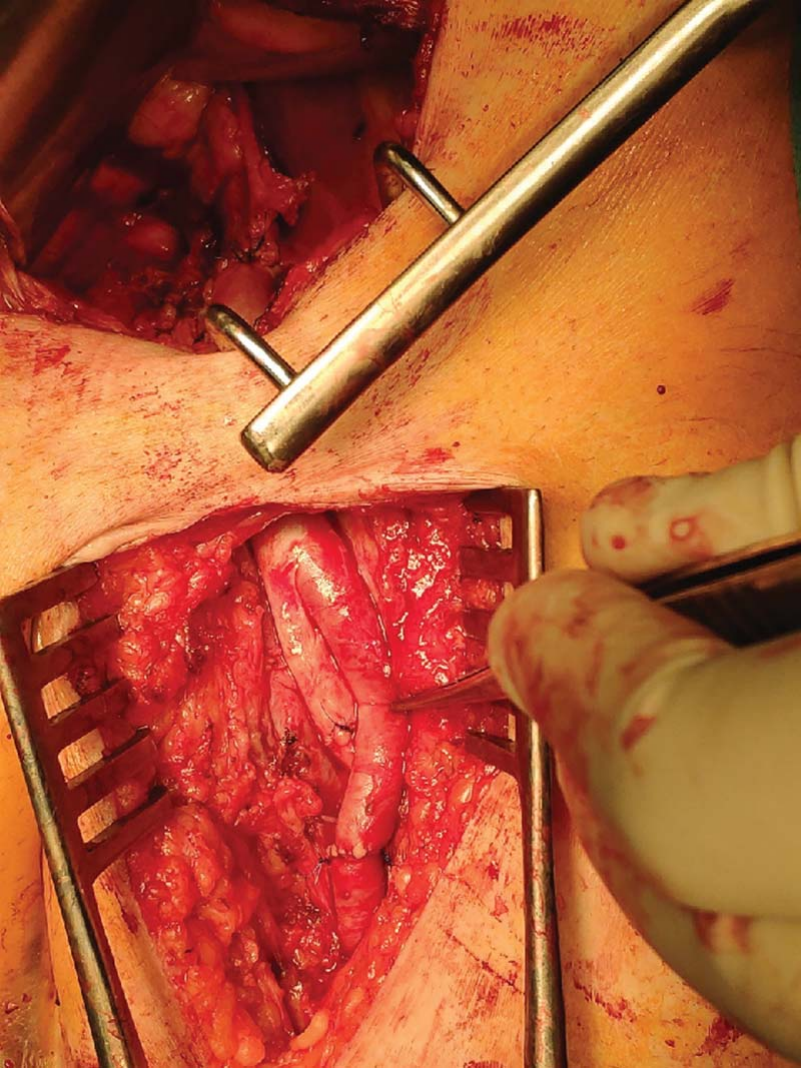
Iliofemoral homograft by both retroperitoneal and inguinal approaches.

